# Robust Angle Estimation for MIMO Radar with the Coexistence of Mutual Coupling and Colored Noise

**DOI:** 10.3390/s18030832

**Published:** 2018-03-09

**Authors:** Junxiang Wang, Xianpeng Wang, Dingjie Xu, Guoan Bi

**Affiliations:** 1 College of Automation, Harbin Engineering University, Harbin 150001, China; wangjunxiang@hrbeu.edu.cn; 2 State Key Laboratory of Marine Resource Utilization in South China Sea, Hainan University, Haikou 570228, China; 3 College of Information Science and Technology, Hainan University, Haikou 570228, China; 4 College of Electical Engineering and Automation , Harbin Institute of Technology University, Harbin 150001, China; xdj1966@hit.edu.cn; 5 School of Electrical and Electronic Engineering, Nanyang Technological University, Singapore 639798, Singapore; egbi@ntu.edu.sg

**Keywords:** MIMO radar, angle estimation, mutual coupling, spatial colored noise, HOSVD

## Abstract

This paper deals with joint estimation of direction-of-departure (DOD) and direction-of- arrival (DOA) in bistatic multiple-input multiple-output (MIMO) radar with the coexistence of unknown mutual coupling and spatial colored noise by developing a novel robust covariance tensor-based angle estimation method. In the proposed method, a third-order tensor is firstly formulated for capturing the multidimensional nature of the received data. Then taking advantage of the temporal uncorrelated characteristic of colored noise and the banded complex symmetric Toeplitz structure of the mutual coupling matrices, a novel fourth-order covariance tensor is constructed for eliminating the influence of both spatial colored noise and mutual coupling. After a robust signal subspace estimation is obtained by using the higher-order singular value decomposition (HOSVD) technique, the rotational invariance technique is applied to achieve the DODs and DOAs. Compared with the existing HOSVD-based subspace methods, the proposed method can provide superior angle estimation performance and automatically jointly perform the DODs and DOAs. Results from numerical experiments are presented to verify the effectiveness of the proposed method.

## 1. Introduction

As an innovative radar framework, multiple-input multiple-output (MIMO) radar system plays an important role in improving detection and estimation performance. MIMO radar uses multiple antennas to emit mutual orthogonal waveforms, and attempts to detect the locations of interest targets from the echoes received by multiple antennas. By exploiting matched filters, the information from an individual transmitter-to-receiver path can be extracted, and a virtual array with a large aperture is formed. Thus, MIMO radar can achieve superior performance compared with the traditional phase-array radar [1]. In terms of the antennas configuration, MIMO radar can be generally divided into two classes: statistical MIMO radar and collocated MIMO radar [2,3]. The former can deal with the scintillation problem by taking advantage of widely separated antennas and the latter can achieve unambiguous angle estimation with closely placed antenna arrays. In this paper, we focus on the bistatic MIMO radar, which is a kind of the collocated MIMO radar.

In bistatic MIMO radar, joint estimation of direction-of-departure (DOD) and direction-of-arrival (DOA) is a key issue. Many excellent algorithms, such as spatial spectrum searching methods [4,5], estimate of signal parameter via rotational invariance techniques (ESPRIT) based methods [6,7], propagator methods (PM) [8,9,10], optimization methods [11,12] and tensor methods [13,14,15], have been investigated. As suggested in these literatures, all the methods can perform well under the desired conditions, such as the well-calibrated transmit and receive arrays, and Gaussian white noise. Nevertheless, in practice, the transmit and receive arrays may suffer from mutual coupling due to the radiation effect from antenna elements [16,17,18,19,20]. On the other hand, the noise model is often unknown spatial colored noise, i.e., the covariance of the noise is no longer a scaled identical matrix. Thus, the high-resolution estimation methods usually have some performance degradation or fail to work. The mutual coupling problem in MIMO radar has been investigated [21,22]. In [23], an improved multiple signal classification (MUSIC) algorithm was proposed to transform the estimation of DOD, DOA and mutual coupling matrices into a linear constrained quadratic problem. However, it is computationally inefficient since two one-dimensional peaking searches are required, and angle ambiguity would appear because the columns in the direction matrices may be linearly dependent. By using the the banded complex symmetric Toeplitz structure in [24], the ESPRIT-Like algorithm was introduced to eliminate the effect of mutual coupling for a closed solution to angle estimation, which avoids the peaking search. A PM-Like method in [25] avoided requiring eigenvalue decomposition (EVD) of the array covariance matrix. Compared with ESPRIT-Like algorithm, it provides similar performance with lower computational complexity. The above matrix-based algorithms ignore the multidimensional nature that is inherent in the received data. Taking the multidimensional structure of the received data into consideration, the higher-order singular value decomposition (HOSVD) method and the trilinear decomposition method were derived in [26,27], respectively to achieve better performance than the conventional subspace methods. On the other hand, efforts have been devoted to solve the angle estimation problem in spatial colored noise environment. For instance, the spatial cross-correlation methods [28,29,30,31], the temporal cross-correlation methods [32,33] and the differencing covariance method [34] have been reported. Although these methods can achieve satisfied performance with spatial colored noise, their performance may have obvious degradation or fail to work. The above mentioned angle estimation methods investigate the mutual coupling and spatial colored noise independently.

In this paper, we attempt to estimate the DODs and DOAs in the environment of mutual coupling and spatial colored noise. To the best knowledge of the authors, there has been little attention to this case in the open literature. Towards this end, a novel robust covariance tensor-based angle estimation method is developed, which can be viewed as a generalized HOSVD approach. The main contribution of this paper can be summarized as: (1) Unlike solving the influence of mutual coupling and spatial colored noise independently in [26,27,28,29,30,31,32,33,34], the proposed method can solve the joint DOD and DOA estimation issue with the coexistence of unknown mutual coupling and spatial colored noise in MIMO radar for the first time. (2) The proposed method formulates a novel fourth-order covariance tensor for eliminating the influence of both spatial colored noise and mutual coupling in tensor domain. (3) The rotational invariance technique is utilized to obtain automatically paired DODs and DOAs. (4) The proposed method provides better angle estimation performance than the existing HOSVD-based subspace methods, and simulation results are performed to show the advantages of the proposed algorithm .

The paper outline is as follows. Some necessary tensor preliminaries and the signal model are given in Section 2. The proposed scheme is established in Section 3. Some remarks and detailed analysis of the proposed algorithm are discussed in Section 4. Simulation results are given in Section 5. Finally, we give the conclusions of the proposed method in Section 6.

Notation: The bold face capital letter X and lower case x denote matrices and vectors, respectively. The M×M identity matrix is denoted by $I_{M}$, and a M×N zero matrix (all the elements are zeros) is denoted by $0_{M\times N}$. For X, the expressions, ${\left(X\right)}^{T}$,${\left(X\right)}^{H}$,${\left(X\right)}^{-1}$ and ${\left(X\right)}^{†}$, represent the operations of transpose, Hermitian transpose, inverse and pseudo-inverse, respectively and ⊗ stands for the Kronecker product. The Khatri-Rao product (column-wise Kronecker product) is denoted by ⊙, i.e., $\left[a_{1},a_{2},\ldots ,a_{K}\right]⊙\left[b_{1},b_{2},\ldots ,b_{K}\right]=\left[a_{1}⊗b_{1},a_{2}⊗b_{2},\ldots ,a_{K}⊗b_{K}\right]$; Toeplitz[r] denotes the symmetric Toeplitz matrix constructed by the vector r; diag(r) denotes the diagonalization operation; angle(·) returns the phase angles of an element in radians.

## 2. Tensor Preliminaries and Signal Model

### 2.1. Tensor Preliminaries

Let us briefly review some necessary operations in tensor domain. For more details about tensor algebra, the readers can refer to the review article [35].

**Definition** **1** (Unfolding or Matricization). *An $\left(I_{1}\times I_{2}\times \cdots \times I_{N}\right)$-dimensional tensor has N indices. The mode-n unfolding of a tensor $X\in C^{I_{1}\times I_{2}\times \cdots \times I_{N}}$ is denoted by ${\left[X\right]}_{\left(n\right)}$, where the $\left(i_{1},i_{2},\ldots ,i_{N}\right)$-element of X maps to the $\left(i_{n},j\right)$-th element of ${\left[X\right]}_{\left(n\right)}$, where $j=1+\sum_{k=1,k\neq n}^{N}\left(i_{k}-1\right)J_{k}$ with $J_{k}=\prod_{m=1,m\neq n}^{k-1}I_{m}$.*

**Definition** **2** (Mode-n Tensor-Matrix Product). *The mode-n product of an N-order tensor $X\in C^{I_{1}\times I_{2}\times \cdots \times I_{N}}$ and a matrix $A\in C^{J_{n}\times I_{n}}$, is denoted by $Y=X_{\times n}A$. In the matrix form, it can be expressed as*

(1) $${\left[Y\right]}_{\left(n\right)}=A{\left[X\right]}_{\left(n\right)}$$

*Moreover, the mode-n tensor-matrix product satisfies the following properties*

(2) $$\left\{\begin{array}{c} X_{\times n}A_{\times m}B=X_{\times m}B_{\times n}A,\;m\neq n\\ X_{\times n}A_{\times n}B=X_{\times n}\left(BA\right),\;m=n \end{array}\right.$$

(3) $${\left[X_{\times 1}A_{1\times 2}A_{2\times }\ldots_{\times N}A_{N}\right]}_{\left(n\right)}=A_{n}\cdot {\left[X\right]}_{\left(n\right)}\cdot \left[A_{n+1}⊗\cdots ⊗A_{N}⊗A_{1}\cdots ⊗A_{n-1}\right]$$

**Definition** **3** (Tensor Decomposition). *The HOSVD of a tensor $X\in C^{I_{1}\times I_{2}\times \cdots \times I_{N}}$ is given by*

(4) $$X=G_{\times 1}U_{1\times 2}U_{2\times 3}\ldots_{\times N}U_{N}$$

*where $G\in C^{I_{1}\times I_{2}\times \cdots \times I_{N}}$ is the core tensor, and $U_{n}\in C^{I_{n}\times I_{n}}(n=1,2,3,\ldots ,N)$ is a unitary matrix, which is consist of the left singular vectors of ${\left[X\right]}_{\left(n\right)}$.*

### 2.2. Signal Model

**Figure 1:**
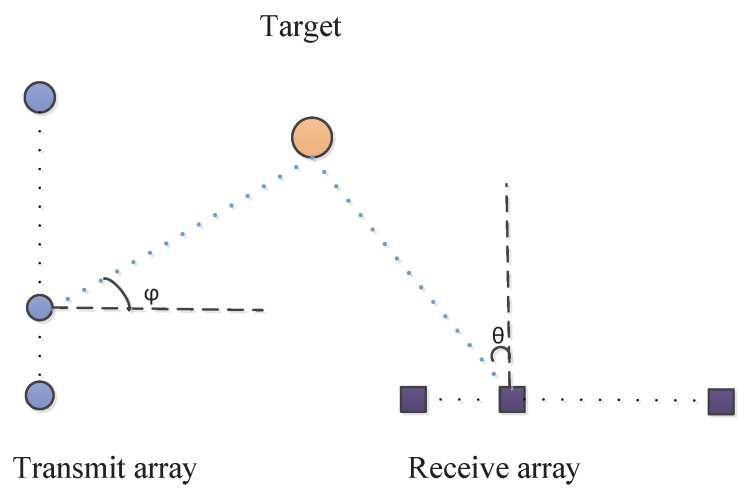
Bistatic MIMO radar configuration.

Now we consider a general bistatic MIMO radar system, as shown in Figure 1. There are *M* transmitters and *N* receivers, and both of which are uniform linear arrays (ULAs) with half-wavelength spacing. The transmitters emit *M* mutual orthogonal waveforms and illuminate a given area. Suppose that there are *K* point targets located in the far field. The signals are reflected by the slow-moving targets and the echoes are collected by the receivers. With the assumtion that each pulse period of the transmitted waveform is consist of *Q* coded symbols, let us consider a coherent processing interval (CPI) consisting of *L* pulses. Then the received data during the *l*-th (l=1,2,…,L) pulse period can be expressed as

(5) $$X_{l}=A_{r}\mathrm{diag}\left(s_{l}\right)A_{t}^{T}W+F_{l}$$

where $A_{r}\in C^{N\times K}$ is the receive direction matrix, $A_{t}\in C^{M\times K}$ is the transmit direction matrix, $s_{l}\in C^{K\times 1}$ is the echo coefficient vector, $W\in C^{M\times Q}$ is the baseband code matrix, and $F_{l}\in C^{N\times Q}$ is the received spatial colored noise. The details of the above mentioned matrices are given below

$$\begin{array}{c} A_{r}=\left[a_{r}\left(\theta_{1}\right),a_{r}\left(\theta_{2}\right),\ldots ,a_{r}\left(\theta_{K}\right)\right]\\ a_{r}\left(\theta_{k}\right)={\left[1,e^{-j\pi \sin\theta_{k}},\ldots ,e^{-j\pi \left(N-1\right)\sin\theta_{k}}\right]}^{T},\;k=1,2,\ldots ,K\\ A_{t}=\left[a_{t}\left(\varphi_{1}\right),a_{t}\left(\varphi_{2}\right),\ldots ,a_{t}\left(\varphi_{K}\right)\right]\\ a_{t}\left(\varphi_{k}\right)={\left[1,e^{-j\pi \sin\varphi_{k}},\ldots ,e^{-j\pi \left(M-1\right)\sin\varphi_{k}}\right]}^{T},\;k=1,2,\ldots ,K\\ s_{l}={\left[\alpha_{1}e^{j2\pi lf_{1}/f_{s}},\alpha_{2}e^{j2\pi lf_{2}/f_{s}},\ldots ,\alpha_{K}e^{j2\pi lf_{K}/f_{s}}\right]}^{T}\\ W=\left[w_{1},w_{2},\ldots ,w_{M}\right] \end{array}$$

where $a_{r}\left(\theta_{k}\right)$ and $a_{t}\left(\varphi_{k}\right)$ denote the receive steering vector and the transmit steering vector, respectively; $\alpha_{k}$, $f_{k}$ and $f_{s}$ represent the radar cross section (RCS) amplitude, the Doppler frequency and the pulse repeat frequency, respectively and $w_{m}\in C^{Q\times 1}$ is the *m*-th transmitted baseband code with $w_{m}^{H}w_{n}=\left\{\begin{array}{c} 0,m\neq n\\ P,m=n \end{array}\right.m,n\in \left\{1,2,\ldots ,M\right\}$. The received noise is spatially colored, which indicates that the columns of $F_{l}$ are independently and identically distributed complex Gaussian random vectors with zero mean and unknown covariance matrix C, i.e., for any $a,b\in \left\{1,2,\ldots ,L\right\}$, there exist

(6) $$E\left\{\mathrm{vec}\left(F_{a}\right)\mathrm{vec}\left(F_{b}^{H}\right)\right\}=\left\{\begin{array}{cc} 0, & a\neq b\\ I_{Q}⊗C, & a=b \end{array}\right.$$

0.98,0.00,0.00 where $F_{a}$ and $F_{b}$ are the columns of $F_{l}$. At the receive array, the received data $X_{l}$ is matched by $w_{m}/Q$, m=1,2,…,M, respectively. After this operation, the matched data can be stacked in to a matrix $\tilde{Y}$ according to the spatial-temporal order. Similar to [24], we have

(7) $$\tilde{Y}=\left[A_{t}⊙A_{r}\right]S^{T}+\frac{1}{Q}N$$

where $S={\left[s_{1},s_{2},\ldots ,s_{L}\right]}^{T}$ is the echo coefficient matrix and $N=\left[n_{1},n_{2},\ldots ,n_{L}\right]$ denotes the matched noise matrix with $n_{l}=\mathrm{vec}\left(F_{l}W^{H}\right)$, (l=1,2,…,L). Generally speaking, mutual coupling effect would exist in the antenna array due to the radiation effects of the antenna elements [16]. The mutual coupling between antenna elements of a ULA can be described as a banded symmetric Toeplitz matrix, known as the mutual coupling matrix. The mutual coupling coefficient between two antennas in a ULA is opposite to their distance and is approximated as zeros when the distance is large enough [24]. Assuming that the number of nonzero mutual coupling coefficients is P+1 for both transmit and receive arrays, and *P* is satisfied with min{M,N}>2P. Taking the mutual coupling effect into consideration, the data model in Equation (7) becomes

(8) $$Y=\left[\left(C_{t}A_{t}\right)⊙\left(C_{r}A_{r}\right)\right]S^{T}+\frac{1}{Q}N=\left[{\tilde{A}}_{t}⊙{\tilde{A}}_{r}\right]S^{T}+\frac{1}{Q}N$$

where $C_{t}=\mathrm{toeplitz}\left(\left[c_{t}^{T},0_{1\times \left(M-P-1\right)}\right]\right)\in C^{M\times M}$ and $C_{r}=\mathrm{toeplitz}\left(\left[c_{r}^{T},0_{1\times \left(N-P-1\right)}\right]\right)\in C^{N\times N}$ are the mutual coupling matrices with $c_{t}=\left[c_{t0},c_{t1},\ldots ,c_{tP}\right]$ and $c_{r}=\left[c_{r0},c_{r1},\ldots ,c_{rP}\right]$, and $c_{ip}\left(i\;=\;r,t;p\;=\;0,1,2,\ldots ,P\right)$ is the P+1 nonzero mutual coupling coefficients, which satisfy with $0<|c_{iP}\left|<,\ldots ,<\right|c_{i1}\left|<\right|c_{i0}|=1$. ${\tilde{A}}_{t}=\left[{\tilde{a}}_{t}\left(\varphi_{1}\right),{\tilde{a}}_{t}\left(\varphi_{2}\right),\ldots ,{\tilde{a}}_{t}\left(\varphi_{K}\right)\right]=C_{t}A_{t}$, ${\tilde{A}}_{r}\;=\;\left[{\tilde{a}}_{r}\left(\varphi_{1}\right),{\tilde{a}}_{r}\left(\varphi_{2}\right),\ldots ,{\tilde{a}}_{r}\left(\varphi_{K}\right)\right]=C_{r}A_{r}$.

**Figure 2:**
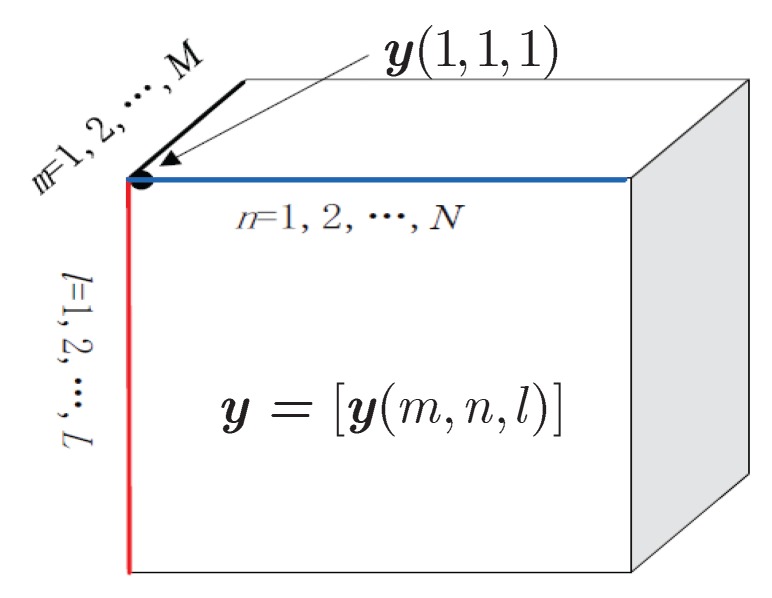
The tensor diagram of received data.

Since each element in Y is formed by multiplying the unique entity in ${\tilde{A}}_{t}$, ${\tilde{A}}_{r}$ and S, the matched data Y exhibits three diversities. However, these diversities have been ignored by the traditional matrix-based estimation methods. To further exploiting the inherent multidimensional structure, Y is rearranged into a third-order tensor Y shown as Figure 2. Then the (m,n,l)-th (m=1,…,M;n=1,…,N;l=1,…,L) element is given by

(9) $$\begin{array}{c} Y\left(m,n,l\right)=\sum_{k=1}^{K}{\tilde{A}}_{t}\left(m,k\right){\tilde{A}}_{r}\left(n,k\right)S\left(l,k\right)+\frac{1}{Q}N\left(m,n,l\right) \end{array}$$

where $Y\left(m,n,l\right)$ denotes the $\left(m,n,l\right)$-th element in Y and similar to others, and $N\in C^{M\times N\times L}$ is the rearranged noise tensor. According to Definition 1, it is obvious that $Y={\left[Y\right]}_{\left(3\right)}^{T}$ and $N={\left[N\right]}_{\left(3\right)}^{T}$. In HOSVD-wise format, Equation (9) can be expressed as

(10) $$Y=I_{K\times 1}{\tilde{A}}_{r\times 2}{\tilde{A}}_{t\times 3}S+\frac{1}{Q}N$$

where $I_{K}$ is the K×K×K identity tensor.

## 3. Robust Covariance Tensor-based Angle Estimation Method

Because the conventional subspace-based methods or tensor decomposition technique [24,25,26,27,28,29,31,33,34] considers the influence of mutual coupling and spatial colored noise independently, they unavoidably have performance degradation or fail to work with the coexistence of unknown mutual coupling and spatial colored noise. Let us formulate a novel robust covariance tensor-based angle estimation method to remove the influence of both mutual coupling and spatial colored noise in tensor domain.

### 3.1. Spatial Colored Noise Suppression and Mutual Coupling Elimination

In Equation (10), the mutual coupling has an impacts on the transmit direction matrix ${\tilde{A}}_{t}$ and receive direction matrix ${\tilde{A}}_{r}$, i.e, ${\tilde{A}}_{t}$ and ${\tilde{A}}_{r}$ are no longer Vandermonde matrices. Fortunately, the mutual coupling matrices are banded symmetric Toeplitz, and two sub-matrices can be extracted from transmit and receive direction matrices for decoupling. By defining two selection matrices as $J_{3}=\left[0_{\left(N-2P\right)\times P},I_{\left(N-2P\right)},0_{\left(N-2P\right)\times P}\right]$ and $J_{4}=\left[0_{\left(M-2P\right)\times P},I_{\left(M-2P\right)},0_{\left(M-2P\right)\times P}\right]$, we have

(11) $$\left\{\begin{array}{c} {\hat{a}}_{r}\left(\theta_{k}\right)=J_{3}{\tilde{a}}_{r}\left(\theta_{k}\right)=\beta_{rk}{\bar{a}}_{r}\left(\theta_{k}\right)\\ {\hat{a}}_{t}\left(\theta_{k}\right)=J_{4}{\tilde{a}}_{t}\left(\varphi_{k}\right)=\beta_{tk}{\bar{a}}_{t}\left(\varphi_{k}\right) \end{array}\right.$$

where $\beta_{tk}=1+\sum_{p=1}^{P}2c_{tp}\cos\left(p\pi \sin\varphi_{k}\right)$, $\beta_{rk}=1+\sum_{p=1}^{P}2c_{rp}\cos\left(p\pi \sin\theta_{k}\right)$, ${\bar{a}}_{r}\left(\theta_{k}\right)$ and ${\bar{a}}_{t}\left(\varphi_{k}\right)$ are column vectors composed of the first N−2P and M−2P elements of $a_{r}\left(\theta_{k}\right)$ and $a_{t}\left(\varphi_{k}\right)$, respectively. From Equation (11), it is easy to know that for each target, $\beta_{tk}$ and $\beta_{rk}$ are constant, which indicates that the direction matrices ${\hat{A}}_{r}\left(\theta \right)=\left[{\hat{a}}_{r}\left(\theta_{1}\right),{\hat{a}}_{r}\left(\theta_{2}\right),\ldots ,{\hat{a}}_{r}\left(\theta_{K}\right)\right]$ and ${\hat{A}}_{t}\left(\theta \right)=\left[{\hat{a}}_{t}\left(\theta_{1}\right),{\hat{a}}_{t}\left(\theta_{2}\right),\ldots ,{\hat{a}}_{t}\left(\theta_{K}\right)\right]$ have the Vandermonde structure. Thus, the influence of mutual coupling is eliminated after the decoupling operation. The decoupling operation in Equation (11) can be extended to Equation (10) in tensor domain, which shows

(12) $$\hat{Y}=Y_{\times 1}J_{3\times 2}J_{4}=I_{K\times 1}{\hat{A}}_{r\times 2}{\hat{A}}_{t\times 3}S+\frac{1}{Q}\hat{N}$$

where $\hat{N}=N_{\times 1}J_{3\times 2}J_{4}$ is a part of $\hat{N}$, which corresponds to the spatial colored noise of (M−2P)(N−2P) elements after the decoupling operation in matrix domain. According to Equation (12), it can be indicated that the effect of mutual coupling has been eliminated in tensor domain, and the tensor noise $\hat{N}$ also holds the same characteristic with N. Thus, the decoupling in Equation (12) also hold the influence of spatial colored noise, which makes the performance degradation. In order to further eliminate the spatial colored noise, it is necessary to analysis the covariance of the colored noise. Let $a,b\in \left\{1,2,\ldots ,L\right\}$, we get

(13) $$\begin{array}{ccc} E\left\{n_{a}n_{b}^{H}\right\} & = & E\left\{\mathrm{vec}\left(F_{a}W^{H}\right){\mathrm{vec}}^{H}\left(F_{b}W^{H}\right)\right\}=E\left\{\mathrm{vec}\left(I_{N}F_{a}W^{H}\right){\mathrm{vec}}^{H}\left(I_{N}F_{b}W^{H}\right)\right\}\\ & = & E\left\{\left[W^{*}⊗I_{N}\right]\left[\mathrm{vec}\left(F_{a}\right){\mathrm{vec}}^{H}\left(F_{b}\right)\right]\left[W^{T}⊗I_{N}\right]\right\}\\ & = & \left\{ \begin{array}{cc} 0, & a\neq b\\ E\left[W^{*}⊗I_{N}\right]\left[I_{M}⊗C\right]\left[W^{T}⊗I_{N}\right], & a=b \end{array}\right.\\ & = & \left\{ \begin{array}{cc} 0, & a\neq b\\ Q(I_{M}⊗C), & a=b \end{array}\right. \end{array}$$

Based on Equation (13), it can be concluded that the covariance matrix of the spatial colored noise with different pulse period is 0, i.e., the spatial colored noise is temporal uncorrelated. Motivated by this feature, the tensor $\hat{Y}$ is divided into two sub-tensors $Z_{1}$ and $Z_{2}$, which are shown as

(14) $$\begin{array}{c} Z_{1}={\hat{Y}}_{\times 3}J_{3}=I_{K\times 1}{\hat{A}}_{r\times 2}{\hat{A}}_{t\times 3}S_{1}+\frac{1}{Q}{\underline{N}}_{1}\\ Z_{2}={\hat{Y}}_{\times 3}J_{4}=I_{K\times 1}{\hat{A}}_{r\times 2}{\hat{A}}_{t\times 3}S_{2}+\frac{1}{Q}{\underline{N}}_{2} \end{array}$$

where $J_{3}=\left[I_{L-1},0_{L-1\times 1}\right]$, $J_{4}=\left[0_{L-1\times 1},I_{L-1}\right]$, $S_{1}=J_{3}S$, ${\underline{N}}_{1}={\hat{N}}_{\times 3}J_{3}$, $S_{2}=J_{4}S$, and ${\underline{N}}_{2}={\hat{N}}_{\times 3}J_{4}$. According to the characteristic of the spatial colored noise in Equation (13), we have

(15) $$R_{Z}=E\left\{{\left[Z_{1}\right]}_{\left(3\right)}^{T}{\left[Z_{2}\right]}_{\left(3\right)}^{*}\right\}=\left[{\hat{A}}_{t}⊙{\hat{A}}_{r}\right]R_{\hat{S}}{\left[{\hat{A}}_{t}⊙{\hat{A}}_{r}\right]}^{H}+\frac{1}{Q^{2}}R_{\underline{N}}=\left[{\hat{A}}_{t}⊙{\hat{A}}_{r}\right]R_{\hat{S}}{\left[{\hat{A}}_{t}⊙{\hat{A}}_{r}\right]}^{H}$$

where $R_{\hat{S}}=E\left[S_{1}^{T}S_{2}^{*}\right]$, and according to Equation (13), $R_{\underline{N}}=E\left\{{\left[{\underline{N}}_{1}\right]}_{\left(3\right)}^{T}{\left[{\underline{N}}_{2}\right]}_{\left(3\right)}^{*}\right\}=E\left\{\left[{\hat{n}}_{1},{\hat{n}}_{2},\ldots ,{\hat{n}}_{L-1}\right]{\left[{\hat{n}}_{2},{\hat{n}}_{3},\ldots ,{\hat{n}}_{L}\right]}^{H}\right\}=0$, which indicates that the spatial colored noise is eliminated. Thus, similar to [26], the de-noising method in Equation (15) can be extended in tensor format. Using the characteristic of the spatial colored noise, a new fourth-order tensor $R\in C^{N\times M\times N\times M}$ is constructed with the (n,m,q,p)-th ($n,q\in \left\{1,2,\ldots ,N-2P\right\};m,p\in \left\{1,2,\ldots ,M-2P\right\}$) element as

(16) $$R\left(n,m,q,p\right)=\sum_{l=1}^{L-1}Z_{1}\left(n,m,l\right)Z_{2}^{*}\left(q,p,l\right)$$

According to the structure of R, the relationship between $R_{Z}$ and R can be written as

(17) $$R_{Z}=\left[\begin{array}{ccccccc} R\left(1,1,1,1\right) & R\left(1,1,2,1\right) & \ldots & R\left(1,1,N,1\right) & R\left(1,1,1,2\right) & \ldots & R\left(1,1,N,M\right)\\ R\left(2,1,1,1\right) & R\left(2,1,2,1\right) & \ldots & R\left(2,1,N,1\right) & R\left(2,1,1,2\right) & \ldots & R\left(2,1,N,M\right)\\ \vdots & \vdots & \ddots & \vdots & \vdots & \ddots & \vdots \\ R\left(N,1,1,1\right) & R\left(N,1,2,1\right) & \ldots & R\left(N,1,N,1\right) & R\left(N,1,1,2\right) & \ldots & R\left(N,1,N,M\right)\\ R\left(1,2,1,1\right) & R\left(1,2,2,1\right) & \ldots & R\left(1,2,N,1\right) & R\left(1,2,1,2\right) & \ldots & R\left(1,2,N,M\right)\\ R\left(2,2,1,1\right) & R\left(2,2,2,1\right) & \ldots & R\left(2,2,N,1\right) & R\left(2,2,1,2\right) & \ldots & R\left(2,2,N,M\right)\\ \vdots & \vdots & \ddots & \vdots & \vdots & \ddots & \vdots \\ R\left(N,M,1,1\right) & R\left(N,M,2,1\right) & \ldots & R\left(N,M,N,1\right) & R\left(N,M,1,2\right) & \ldots & R\left(N,M,N,M\right) \end{array}\right]$$

0.98,0.00,0.00 From Equation (17), it is clearly seen that the spatial colored noise is suppressed in R, which indicates that the covariance tensor R is without the influence of both mutual coupling and spatial colored noise. Similar to the model in Equation (10), R can be rewritten as

(18) $$R=R_{S\times 1}{\hat{A}}_{r\times 2}{\hat{A}}_{t\times 3}{\hat{A}}_{r\times 3}^{*}{\hat{A}}_{t}^{*}$$

where $R_{S}\in C^{K\times K\times K\times K}$ is the Hermitian folding of $R_{\tilde{S}}$ in the same way as Equation (17), i.e., $R_{S}\left(:,k_{1},:,k_{2}\right)=\left\{\begin{array}{c} 0,k_{1}\neq k_{2}\\ R_{\tilde{S}}\left(k_{1},k_{2}\right),k_{1}=k_{2} \end{array}\right.$, $\;k_{1},k_{2}\in \left\{1,2,\ldots ,K\right\}$. The decomposition of fourth-order tensor in Equation (18) is commonly known as the ’Tucker4’ decomposition, where the corresponding core tensor is $R_{S}$.

### 3.2. Tensor-Based Signal Subspace Estimation and Angle Estimation

In order to exploit the subspace technique for angle estimation, the signal/noise subspace is required to estimate firstly. Although the traditional SVD technique is easy to implement for estimating the signal/noise subspace, the multidimensional structure inherent in R is ignored. Fortunately, the HOSVD technique is an effective way to estimate the signal subspace with capturing the multidimensional structure information in tensor. The HOSVD of the measurement R is given by [31]

(19) $$R=G_{\times 1}U_{1\times 2}U_{2\times 3}U_{3\times 4}U_{4}$$

where $G\in C^{\left(N-2P)\times (M-2P)\times (N-2P)\times (M-2P\right)}$ is the associated core tensor, $U_{1}\in C^{\left(N-2P)\times (N-2P\right)}$, $U_{2}\in C^{\left(M-2P)\times (M-2P\right)}$, $U_{3}\in C^{\left(N-2P)\times (N-2P\right)}$, $U_{4}\in C^{\left(M-2P)\times (M-2P\right)}$ are unitary matrices, which are composed of the left singular vectors of the mode-*n* ($n\in \left\{1,2,3,4\right\}$) matrix unfolding of R as ${\left[R\right]}_{\left(n\right)}=U_{n}\Sigma_{n}V_{n}^{H}$. Note that the rank of R is *K*, hence R can be approximated by its truncated HOSVD as

(20) $$R_{s}=G_{s\times 1}U_{1s\times 2}U_{2s\times 3}U_{3s\times 4}U_{4s}$$

where $U_{is}$ (i=1,2,3,4) is consist of the singular vectors corresponding to the *K* dominant singular values of $U_{i}$. Note that R is Hermitian, we have $U_{1s}=U_{3s}^{*}$, $U_{2s}=U_{4s}^{*}$. $G_{s}=R_{\times 1}U_{1s\times 2}^{H}U_{2s\times 3}^{H}U_{3s\times 4}^{H}U_{4s}^{H}$ accounting for the reduced core tensor. Insertion of $G_{s}$ into Equation (20) yields

(21) $$R_{s}=R_{\times 1}{\left(U_{1s}U_{1s}^{H}\right)}_{\times 2}{\left(U_{2s}U_{2s}^{H}\right)}_{\times 3}{\left(U_{1s}U_{1s}^{H}\right)}_{\times 4}^{*}{\left(U_{2s}U_{2s}^{H}\right)}^{*}$$

By Hermitian unfolding of $R_{s}$, a new cross-correlation matrix $\tilde{R}$ is formed to be

(22) $$\hat{R}=\left[\left(U_{1s}U_{1s}^{H}\right)⊗\left(U_{2s}U_{2s}^{H}\right)\right]R_{Z}{\left[\left(U_{1s}U_{1s}^{H}\right)⊗\left(U_{2s}U_{2s}^{H}\right)\right]}^{H}$$

Since the rank of $R_{Z}$ is *K*, it can be approximated by its truncated EVD as $R_{Z}\approx U_{s}\Lambda_{s}U_{s}^{H}$, where $U_{s}$ contains the singular vectors corresponding to the *K*-dominate singular values. Obviously, $U_{s}$ spans the same subspace by the columns of $\left[{\tilde{A}}_{t}⊙{\tilde{A}}_{r}\right]$. Inserting $R_{\tilde{Y}}$ into Equation (22), we get

(23) $$\hat{R}=\left[\left(U_{1s}U_{1s}^{H}\right)⊗\left(U_{2s}U_{2s}^{H}\right)U_{s}\right]\Sigma {\left[\left(U_{1s}U_{1s}^{H}\right)⊗\left(U_{2s}U_{2s}^{H}\right)U_{s}\right]}^{H}$$

After truncating EVD of $R_{s}$, a new signal subspace ${\hat{U}}_{s}$ is obtained as

(24) $${\hat{U}}_{s}=\left[\left(U_{1s}U_{1s}^{H}\right)⊗\left(U_{2s}U_{2s}^{H}\right)\right]U_{s}$$

Equation (24) describes the relationship between the signal subspace obtained via matrix-based method and that estimated via tensor-based method, from which we can observe that $U_{s}$ is weighted by $\left[\left(U_{1s}U_{1s}^{H}\right)⊗\left(U_{2s}U_{2s}^{H}\right)\right]$. Due to the weighting effect, the noise in the tensor measurement is suppressed to obtain an enhanced signal subspace. Obviously, ${\hat{U}}_{s}$ spans the same space as $U_{s}$. Therefore, there exists a full-rank matrix T that

(25) $${\hat{U}}_{s}=\left[{\hat{A}}_{t}⊙{\hat{A}}_{r}\right]T$$

### 3.3. Joint DOD and DOA Estimation

After obtaining the tensor-based signal subspace without the influence of both mutual coupling and spatial colored noise, the conventional ESPRIT method can be applied to estimate the DODs and DOAs [6,7]. Let $\hat{a}={\hat{a}}_{t}\left(\varphi_{k}\right)⊙{\hat{a}}_{r}\left(\theta_{k}\right)$, and define $J_{t1}=\left[I_{\left(M-2P-1\right)},0_{\left(M-2P-1\right)\times 1}\right]$, $J_{t2}=\left[0_{\left(M-2P-1\right)\times 1},I_{\left(M-2P-1\right)}\right]$, $J_{r1}=\left[I_{\left(N-2P-1\right)},0_{\left(N-2P-1\right)\times 1}\right]$ and $J_{r2}=\left[0_{\left(N-2P-1\right)\times 1},I_{\left(N-2P-1\right)}\right]$. Then we can get the following rotational invariance properties

(26) $$\left\{\begin{array}{c} e^{-j\pi \sin\varphi_{k}}\left[J_{t1}⊗I_{N-2P}\right]\hat{a}=\left[J_{t2}⊗I_{N-2P}\right]\hat{a}\\ e^{-j\pi \sin\theta_{k}}\left[I_{M-2P}⊗J_{r1}\right]\hat{a}=\left[I_{M-2P}⊗J_{r2}\right]\hat{a} \end{array}\right.$$

Let $\Phi_{t}=\mathrm{diag}\left\{e^{-j\pi \sin\varphi_{1}}ande^{-j\pi \sin\varphi_{2}},\ldots ,e^{-j\pi \sin\varphi_{K}}\right\}$, $\Phi_{r}=\mathrm{diag}\left\{e^{-j\pi \sin\theta_{1}},e^{-j\pi \sin\theta_{2}},\ldots ,e^{-j\pi \sin\theta_{K}}\right\}$. Combined with Equation (25) and Equation (26), we can get the least squares (LS) estimation of $\Phi_{t}$ and $\Phi_{r}$ via

(27) $$\left\{\begin{array}{c} {\hat{\Phi }}_{t}={\left(\left[J_{t1}⊗I_{N-2P}\right]{\hat{U}}_{s}\right)}^{†}\left[J_{t2}⊗I_{N-2P}\right]{\hat{U}}_{s}\\ {\hat{\Phi }}_{r}={\left(\left[I_{M-2P}⊗J_{r1}\right]{\hat{U}}_{s}\right)}^{†}\left[I_{M-2P}⊗J_{r1}\right]{\hat{U}}_{s} \end{array}\right.$$

where the relations between ${\hat{\Phi }}_{t},\Phi_{t}$ and ${\hat{\Phi }}_{r},\Phi_{r}$ are ${\hat{\Phi }}_{t}=T\Phi_{t}T^{-1}$, and ${\hat{\Phi }}_{r}=T\Phi_{r}T^{-1}$, respectively. Once ${\hat{\Phi }}_{t}$ has been estimated, the estimation $e^{-j\pi \sin{\hat{\varphi }}_{k}}$ can be obtained via the eigenvalue of ${\hat{\Phi }}_{t}$. Let $\hat{T}$ be the estimated eigenvectors of ${\hat{\Phi }}_{t}$, then the estimation $e^{-j\pi \sin{\hat{\theta }}_{k}}$ can be obtained via the diagonal element of $\hat{T}{\hat{\Phi }}_{r}{\hat{P}}^{-1}$. Finally, the *k*-th (k=1,2,…,K) DOD and DOA are given by

(28) $$\left\{\begin{array}{c} {\hat{\varphi }}_{k}=\arcsin\left[-\mathrm{angle}\left(\upsilon_{k}\right)/\pi \right]\\ {\hat{\theta }}_{k}=\arcsin\left[-\mathrm{angle}\left(\nu_{k}\right)/\pi \right] \end{array}\right.$$

Because ${\hat{\Phi }}_{t}$ and ${\hat{\Phi }}_{r}$ share the same eigenvectors, the estimated DODs and DOAs are paired automatically.

The proposed method is summarized as follows.

Step.1 Stack the matched data into a third-order tensor Y as Equation (9);
Step.2 Construct two sub-tensors $Y_{1}$ and $Y_{2}$ through Equation (12) and Equation (14), then form the cross-covariance tensor R through Equation (16);
Step.3 Perform HOSVD of R to get $U_{ns}$, (n=1,2,3,4), and get $\tilde{R}$ via Equation (22);
Step.4 Perform EVD of $R_{s}$ to get ${\tilde{U}}_{s}$, obtain ${\hat{\Phi }}_{t}$ and ${\hat{\Phi }}_{r}$ via Equation (27), and compute EVD of ${\hat{\Phi }}_{t}$ to get $\Phi_{t}$ and $\hat{T}$.
Step.5 Calculate $\Phi_{r}$ via $\hat{T}{\hat{\Phi }}_{r}{\hat{T}}^{-1}$ and finally get the DODs and DOAs via Equation (28).

## 4. Remarks and Algorithm Analysis

### 4.1. Related Remarks

**Remark** **1.** *The estimation algorithms in [24,25,26] are only effective with uniform white noise, while the denoising methods in [28,29,30,31,32,33,34] may suffer from mutual coupling. Consequently, their performances would degrade to deal with the co-existence mutual coupling and spatial colored noise. However, the proposed method can circumvent the limitations above to achieve better performance than these early reported algorithms.*

**Remark** **2.** *The main drawback of the MUSIC-Like algorithm is the ambiguity [23], since the columns in the mutual coupling matrices may be linear dependent. From Equation (12) we can observe that the direction submatrices exhibit a Vandermonde-Like structure to remove the ambiguity in the proposed method.*

**Remark** **3.** *The HOSVD of a tensor is calculated by SVDs of all the unfolded matrices. However, since $U_{1s}=U_{3s}^{*}$, $U_{2s}=U_{4s}^{*}$, we only need to compute the truncated SVDs of the mode-*1* and mode-*2* unfolding of R, resulting in a significant computational saving.*

### 4.2. Computation Complexity

Now we analysis the complexity of the proposed method in terms of the number of complex multiplications. The complexity of forming R is $M^{2}N^{2}\left(L-1\right)$ and performing truncated HOSVD of R requires $2{\left(M-2P\right)}^{3}{\left(N-2P\right)}^{3}$. The computation load of calculating $\hat{R}$ is $2{\left(M-2P\right)}^{2}K+2{\left(N-2P\right)}^{2}K+2{\left(M-2P\right)}^{2}{\left(N-2P\right)}^{2}$. Computing EVD of $\hat{R}$ needs $\left({\left(M-2P\right)}^{3}{\left(N-2P\right)}^{3}\right)$ complex multiplications. The computational complexity of Equation (27) is $2\left(M-2P-1\right)\left(N-2P\right)K^{2}+2\left(N-2P-1\right)\left(M-2P\right)K^{2}+2\left(K^{3}\right)$. Estimating DOD and DOA through ${\hat{\Phi }}_{t}$ and ${\hat{\Phi }}_{r}$ needs $K^{3}$ complex multiplications. I summary, the total complexities of the proposed method, the ESPRIT-Like method [24], the PM-Like method [25] and the HOSVD method [26] are presented in Table 1. Clearly, the complexity of the proposed method is a slight heaver than the HOSVD method, the ESPRIT-Like method and the PM-Like method. However, it provides much better estimation performance than all these other methods, which will be shown in the simulation section.

## 5. Simulation Results

In the simulation, the bistatic MIMO radar is configured with M=10 transmit elements and N=12 receive elements. The transmit code length is Q=256 and the pulse repeat frequency is $f_{s}=20$ KHz. Suppose that there are K=3 uncorrelated targets located at the directions $\left(\theta_{1},\varphi_{1}\right)=\left({-30}^{{}^{\circ}},10^{{}^{\circ}}\right)$, $\left(\theta_{2},\varphi_{2}\right)=\left(20^{{}^{\circ}},0^{{}^{\circ}}\right)$, $\left(\theta_{3},\varphi_{3}\right)=\left(0^{{}^{\circ}},and{-20}^{{}^{\circ}}\right)$. The corresponding RCS are $\alpha_{1}=\alpha_{2}=\alpha_{3}=1$ and the Doppler shifts are ${\left\{f_{k}\right\}}_{k=1}^{3}=\left\{200,400,850\right\}$Hz, respectively. The spatial colored noise is modeled as a second-order autoregressive (AR) process with the coefficient $z=\left[1,-1,0.8\right]$ [31,33]. The SNR in the simulation is defined by $\mathrm{SNR}=10\log_{10}{∥X_{l}-F_{l}∥}^{2}/{∥F_{l}∥}^{2}\left[\mathrm{dB}\right]$, where $X_{l}$ and $F_{l}$ are the matrices in Equation (5). In the following simulations, two measures are applied for performance assessment [33]. The first one is the root mean square error (RMSE), defined as

$$\mathrm{RMSE}=\frac{1}{K}\sum_{k=1}^{K}\sqrt{\frac{1}{\zeta }\sum_{i=1}^{\zeta }\left\{{\left({\hat{\theta }}_{i,k}-\theta_{k}\right)}^{2}+{\left({\hat{\varphi }}_{i,k}-\varphi_{k}\right)}^{2}\right\}}$$

where ${\hat{\theta }}_{i,k}$ and ${\hat{\varphi }}_{i,k}$ represent the estimations of $\theta_{k}$ and $\varphi_{k}$ for the *i* th Monte Carlo trial, respectively. ζ is the total number of Monte Carlo trials. The other one is the probability of the successful detection (PSD) defined as $\mathrm{PSD}=\frac{D}{\zeta }\times 100\%$, where *D* denotes the total numbers of successful trial and a successful trial is recognized if the absolute errors of all the estimated angles are smaller than 0.1°.

**Figure 3:**
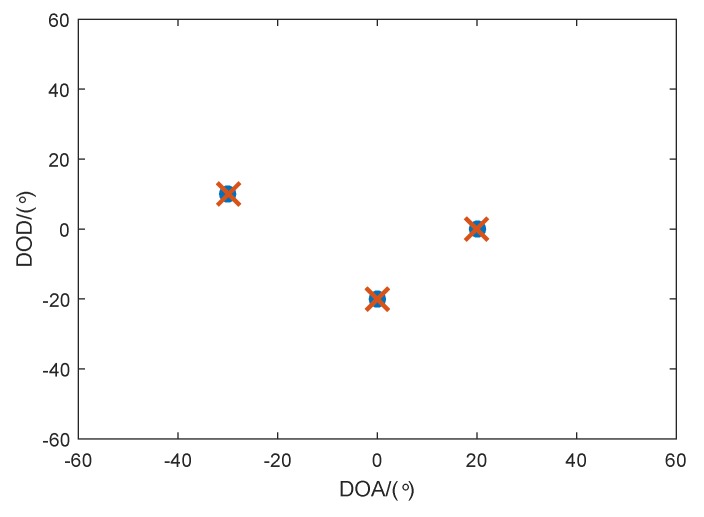
Estimation results of the proposed method with SNR=0dB.

**Figure 4:**
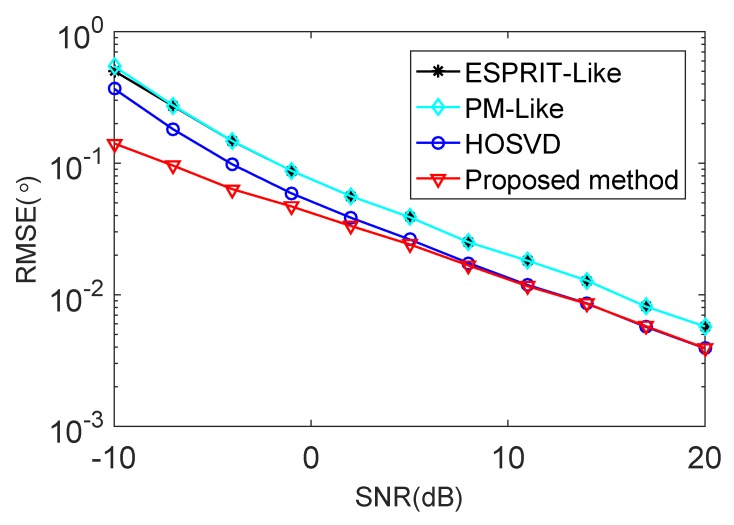
RMSE performance comparison versus SNR in the presence of weak mutual coupling.

**Figure 5:**
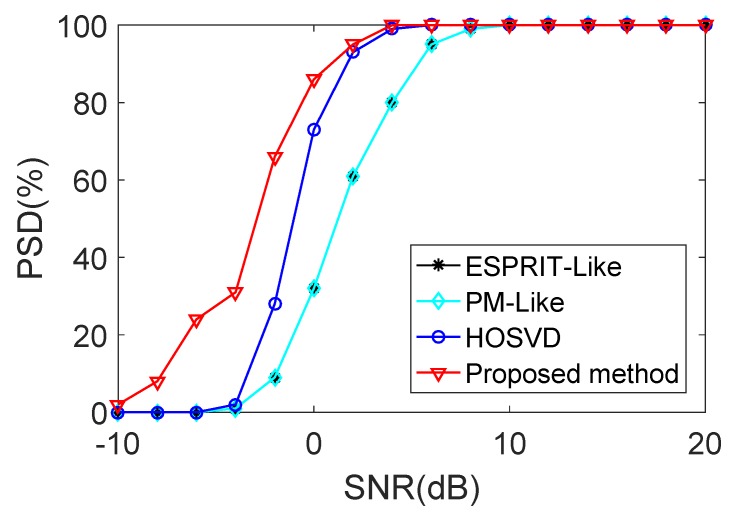
PSD performance comparison versus SNR in the presence of weak mutual coupling.

In the first step simulation results from Figure 3, Figure 4 and Figure 5, we consider the transmit and receive arrays with a weak mutual coupling defined by the mutual coefficients $c_{t}=\left[1,0.1174+j0.0577\right]$ and $c_{r}=\left[1,-0.0121-j0.1029\right]$, respectively. The number of snapshots is 50. Figure 3 illustrates the estimation results of the proposed method with SNR=0dB, from which we can observe that the DODs and DOAs are accurately obtained and correctly paired, which proves the effectiveness of the proposed method.

Figure 4 and Figure 5 show the RMSE and the PSD performance comparisons of the proposed method with the ESPRIT-Like method [24], the PM-Like method [25] and the HOSVD method [26], respectively. It can be concluded from Figure 4 that the proposed method provides much better estimation perfprmance than the ESPRIT-Like method, the PM-Like method and the HOSVD method in the low SNR region. The reason is that the proposed method can eliminate the effect of the mutual coupling and spatially colored noise simultaneously, and the other methods only consider the effect of the mutual coupling. It is shown in Figure 5 that all the methods exhibit a 100% successful detection in the high SNR region. As the SNR decreases, the PSD of each method starts to drop at a certain point, which is defined as the SNR threshold [33]. The proposed method provides lower PSD threshold and better detection performance than all the compared methods.

**Figure 6:**
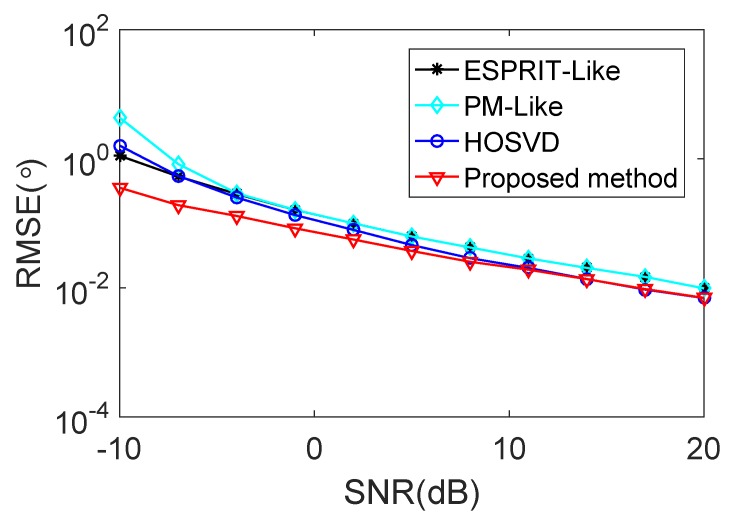
RMSE performance comparison versus SNR with strong mutual coupling.

**Figure 7:**
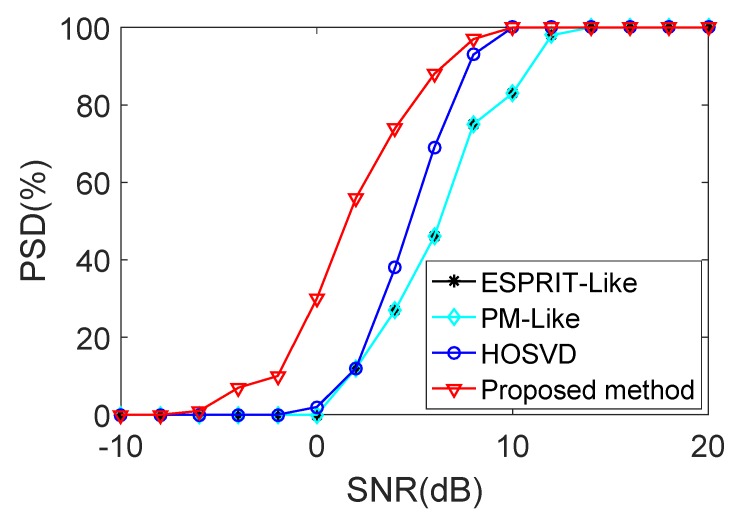
PSD performance comparison versus SNR with strong mutual coupling.

In the second step simulation results shown in Figure 6 to Figure 7, we consider the transmit and receive arrays with a strong mutual coupling, and the mutual coupling coefficients are fixed at $c_{t}=\left[1,0.8+j0.5,0.2+j0.1\right]$, and $c_{r}=\left[1,0.6+j0.4,0.1-j0.3\right]$. The number of snapshots is 50. Figure 6 and Figure 7 illustrate the RMSE and PSD curves of various methods versus SNR. According to the results, the RMSE and PSD performances of all the methods gradually improved with the increasing SNR. However, the ESPRIT-Like method, the PM-Like method and the HOSVD method provide higher RMSE than the proposed method in low SNR region. On the other hand, the proposed method has estimation performance similar to that achieved by the HOSVD method in high SNR region. This is because that the spatially colored noise is not the important factor to effect the estimation performance when the SNR is high enough. From Figure 7, it is obvious that the proposed method has better PSD than the other methods, i.e., it outperforming all the compared methods.

## 6. Conclusions

In this paper, a robust covariance tensor-based angle estimation method is developed for bistatic MIMO radar with the co-existence mutual coupling and spatial colored noise. The proposed method can capture the multidimensional nature of the matched array data. At the same time, it can eliminate the effect of mutual coupling and colored noise in the tensor domain. As a result, the proposed algorithm provides better estimation performance than the existing estimation algorithms. The robustness and superiority of the proposed method are clearly verified by simulation results.

## Figures and Tables

**Table 1:** Comparison of the complexity in joint DOD and DOA estimation.

Method	Computational Complexity
ESPRIT-Like	$\begin{array}{c} {\left(M-2P\right)}^{2}{\left(N-2P\right)}^{2}L+2\left(M-2P-1\right)\left(N-2P\right)K^{2}+2\left(N-2P-1\right)\left(M-2P\right)K^{2}\\ +3K^{3}+2{\left(M-2P\right)}^{3}{\left(N-2P\right)}^{3} \end{array}$
PM-Like	$\begin{array}{c} {\left(M-2P\right)}^{2}{\left(N-2P\right)}^{2}L+2\left(M-2P\right)\left(N-2P\right)K^{2}+2\left(M-2P-1\right)\left(N-2P\right)K^{2}\\ +\left(M-2P\right)\left(N-2P\right)K\left(\left(M-2P\right)\left(N-2P\right)-K\right)+2\left(N-2P-1\right)\left(M-2P\right)K^{2}+3K^{3} \end{array}$
HOSVD	$\begin{array}{c} {\left(M-2P\right)}^{2}{\left(N-2P\right)}^{2}K+0.5\left(M-2P-1\right)\left(N-2P\right)K^{2}\\ +0.5\left(N-2P-1\right)\left(M-2P\right)K^{2}+K^{3}+2{\left(N-2P\right)}^{3}{\left(M-2P\right)}^{3} \end{array}$
Proposed	$\begin{array}{c} M^{2}N^{2}\left(L-1\right)+2{\left(M-2P\right)}^{2}K+2{\left(N-2P\right)}^{2}K+2{\left(M-2P\right)}^{2}{\left(N-2P\right)}^{2}+\\ 2\left(M-2P-1\right)\left(N-2P\right)K^{2}+2\left(N-2P-1\right)\left(M-2P\right)K^{2}+\\ 3K^{3}+3{\left(M-2P\right)}^{3}{\left(N-2P\right)}^{3}+{\left(M-2P\right)}^{3}{\left(N-2P\right)}^{3} \end{array}$
